# Preliminary Water and Sediment Quality Assessment of the Meycauayan River Segment of the Marilao-Meycauayan-Obando River System in Bulacan, the Philippines

**DOI:** 10.5696/2156-9614-10.26.200609

**Published:** 2020-05-26

**Authors:** John Vincent R. Pleto, Veronica P. Migo, Mark Dondi M. Arboleda

**Affiliations:** 1 Environmental Biology Division, College of Arts and Sciences, Institute of Biological Sciences, University of the Philippines Los Baños, Los Baños, Philippines; 2 Department of Chemical Engineering, College of Engineering and Agro-Industrial Technology, University of the Philippines Los Baños, Los Baños, Philippines; 3 School of Environmental Science and Management, University of the Philippines Los Baños, Los Baños, Philippines

**Keywords:** Philippines, Meycauayan River, extreme pollution, organic and heavy metal contamination

## Abstract

**Background.:**

The Meycauayan River is considered one of the most severely polluted rivers in the Philippines due to heavy metal and organic pollution that has caused environmental degradation.

**Objectives.:**

The aim of the present study was to provide insight on the current status of the Meycauayan River and outline an appropriate strategy to solve problems of organic and heavy metal contamination.

**Methods.:**

The physical, chemical and biological characteristics of the water and sediments were analyzed and evaluated based on available local and international standards. Three sites (upstream, midstream and downstream) of the river were considered for the evaluation of water and sediment quality.

**Results.:**

Dissolved oxygen, measured in the morning, was very low at the upstream sampling station (1.87 ppm) and even lower at the downstream site (0.49 ppm). The temperature for the three sites ranged from 28.03°C (upstream) to 30.75°C (downstream). Visual inspection indicated that the color of the water was gray upstream and midstream, and black at the downstream station. Biochemical oxygen demand exceeded the recommended limits of the Department of Environment and Natural Resources (DENR) of 7.0 ppm with values of 13.22 ppm (upstream) and 12.02 ppm (downstream). Chemical oxygen demand exceeded the limit of 20 ppm at the downstream site at 84 ppm. Dissolved oxygen did not reach the recommended limit of 5.0 ppm of the DENR. There was a high coliform count at both the upstream (3.5 × 104 colony-forming unit (cfu)/ml) and downstream (2.5 × 104) sites, which exceeded the limit of the United States Environmental Protection Agency (USEPA) of 126 cfu/100 ml. Heavy metals such lead (Pb), zinc (Zn), copper (Cu), manganese (Mn) and chromium (Cr) exceeded the severe effect level of the National Oceanic and Atmospheric Administration (NOAA), which could be detrimental to humans and aquatic life. The results of one-way analysis of variance showed significant differences (p <0.001) in pH, temperature, dissolved oxygen, conductivity, total dissolved solids, chemical oxygen demand, nitrates and phosphates for water quality and Pb, Zn, Cu, Mn and Cr for sediment quality across the study sites.

**Conclusions.:**

The results of the present study indicate that the downstream site was more polluted, possibly due to the accumulation of pollutants coming from the upstream site. The deterioration of the Meycauayan River is a result of rapid industrialization, urbanization and population growth. Examination of the water quality of the Meycauayan River indicates that it is very polluted and requires an immediate solution. The results of the present study should be used as a basis for crafting strategies to rehabilitate the Meycauayan River.

**Competing Interests.:**

The authors declare no competing financial interests. This study was funded by Pure Earth.

## Introduction

The Meycauayan River segment, in Bulacan, the Philippines, is part of the Marilao-Meycauayan-Obando river system and was included in the 2007 “The Dirty Thirty” list by the Blacksmith Institute/Pure Earth.^1^ The Department of Environment and Natural Resources (DENR)—Environmental Management Bureau categorized the river as Class C, as its beneficial uses include fishery water for propagation and growth of fish, recreational water use (boating) and industrial water supply (for manufacturing processes after treatment).^2^ Heavy metal and organic pollution are severe in this river system and have caused environmental degradation such as decreased quality of fish harvest and degraded water quality for aquaculture ponds. Water pollution can also pose public health problems such as accumulation of heavy metals in fish, which can affect human consumers of fish from this river system through increased blood lead levels of heavy metals, which can be fatal. The river system is heavily polluted due to many industrial and domestic sources, including untreated municipal wastewater from sewers and poorly operated septic tanks; heavy metals (chromium (Cr), lead (Pb), cadmium (Cd), etc.) and chemical discharges from formal and informal industries such as used Pb-acid battery recycling, leather tanning industries, gold and precious metals refining and jewelry making, solid waste dumped into the rivers or drainage, as well as silt, wastes, oil and other contaminants discharged as part of the runoff in the area.^3^

Water pollution due to industrial activities and technological development poses significant threats to the environment and public health because of its toxicity, nonbiodegradability and bioaccumulation. The degraded state of the water resources in the Philippines arises from the absence of an effective system to stop the uncontrolled dumping of untreated sewage, garbage and industrial effluents into water bodies. In light of these problems, it is important to determine and assess the condition of the surrounding environment. A river's water quality is caused by several interrelated compounds which are subjected to spatial and temporal variation and affected by water volume.^4^ Water quality assessment is an important ecological investigation to determine the current condition of a river. This preliminary study can serve as a basis for the crafting of strategies to revive, rehabilitate, and protect the river system in Bulacan.

The general objective of the present study was to assess the physico-chemical, microbiological and sediment quality of the river system. The study specifically aimed to examine physico-chemical water quality variables (dissolved oxygen, temperature, pH, conductivity, total dissolved solids, nitrate, phosphate, and biochemical and chemical oxygen demand) and biological quality (total bacterial and total coliform count) of the Meycauayan River. In addition, the present study aimed to determine the heavy metal content of the river sediment and identify the site with the highest pollution load in the river system.

**Abbreviations**
ANOVA: Analysis of variance
cfu: Colony-forming unit
DENR: Department of Environment and Natural Resources
NOAA: National Oceanographic and Atmospheric Administration
ORP: Oxidation reduction potential
TDS: Total dissolved solids

## Methods

**Figure 1:**
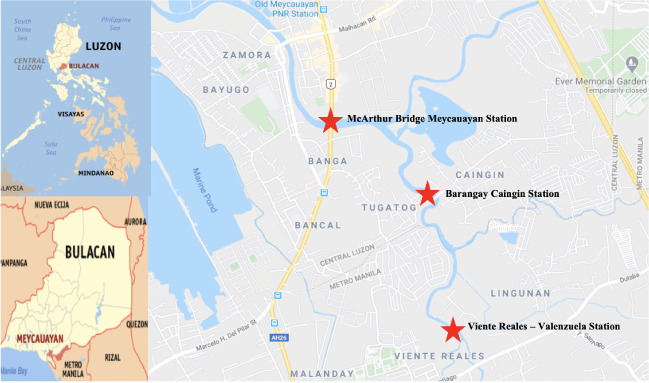
Location map of Meycauayan Bulacan and location of the sampling stations. (Source: Google Maps)

The study area was the Meycauayan River segment of the Marilao-Meycauayan-Obando river system in Bulacan. Bulacan is located immediately north of the National Capital Region. Three sampling points were chosen to be representative of the river. The sampling sites were chosen to determine which portion had the highest pollution level. The upstream site was located at Viente Reales, Valenzuela City. The midstream site was located at Barangay Caingin, Meycauayan and the downstream site was at McArthur Bridge, Meycauayan, Bulacan. The sampling stations were monitored for water quality assessment by the DENR-Environment Management Bureau. Figure 1 presents a map of the study area.

### Water sample collection

Water samples were collected from upstream, midstream and downstream locations of the river segment on October 10, 2014 from 9:00 am to 10:00 am. Four replicate samples were obtained for the determination of the *in-situ* water quality indicators. Four replicate samples were obtained and pooled equally into two 1 L polyethylene bottles for analysis. The samples were placed in an ice chest for transport to the Central Analytical Services Laboratory, BIOTECH, University of the Philippines Los Baños for the *ex-situ* analysis.

### Water quality determination and analysis

Table 1 presents the analytical methods and instruments used for the determination of the different variables.

**Table 1:** Analytical Methods Used for Determinations of Water Quality Variables

**Variables**	**Instruments used/analytical method**
*In-situ* determination	
Dissolved oxygen	HACH HQ30d Portable Dissolved Oxygen Meter
pH	Myron L Ultrameter III 9P
Temperature	Myron L Ultrameter III 9P
Conductivity	Myron L Ultrameter III 9P
Total dissolved solids	Myron L Ultrameter III 9P
Oxidation reduction potential	Myron L Ultrameter III 9P
*Ex-situ* determination	
Nitrate	Brucine Method^5^
Phosphate	Ascorbic Acid Method^6^
Biochemical oxygen demand	5-day Method/Winkler Method^7^
Chemical oxygen demand	Open Reflux Method^8^
Total bacterial count	Standard Plate Count Method^9^
Total coliform count	Standard Coliform Plate Count Method^9^

### Total bacterial and total coliform count

Four replicate samples were obtained and pooled equally into one sterilized dilution bottle. The dilution bottle was placed in an ice chest during transport to the laboratory. Serial dilutions of the water sample were prepared. Each dilution was plated on three replicates of solidified freshly prepared nutrient agar and violet red bile agar (for total coliform count) and spread using a sterile glass rod and incubated at room temperature (30°C) for 24 hours after which the colonies that developed on the plates were counted. Those counts with 25–250 colony-forming units (cfu) were reported as total viable count.^9^

### Sediment collection and analysis

Surface sediments were collected at each site along the river using a grab sampler. Replicate samples were collected and pooled to represent the site. Samples were brought to the laboratory for heavy metal analysis. Prior to analysis, the samples were air dried for 24–48 hours. After air drying, the sediments were analyzed using X-ray fluorescence spectrometry (Niton-XRF analyzer).

### Data and statistical analysis

The water quality indicators at the upstream and downstream sites were compared and analyzed. Physico-chemical and microbiological results were compared to different water quality standards for freshwater to determine whether they were within the recommended safe limits based on the DENR administrative order 2016–08.^10^ Sediment quality was assessed using the standard set by the National Oceanographic and Atmospheric Administration (NOAA) for sediment quality.^11^ One-way analysis of variance (ANOVA) and post-hoc Tukey's test were used to determine significant differences across the sites for the study variables.

## Results

The mean physico-chemical variables and water quality criteria from different agencies are presented in Table 2. The visual color of the water at the upstream site was gray, while water at the downstream site appeared black. The water was slightly alkaline in nature as indicated by its pH. The mean pH of the upstream site was 7.49, while the midstream and downstream sites had a pH of 7.28 and 7.35, respectively. According to DENR 2016–08, it was within the recommended limits of 6.5–9.0.^10^ The temperature from upstream to downstream was within the recommended range of 25–31°C set by the DENR during the sampling time. The dissolved oxygen levels at the three sites were very low and did not meet the standard level of 5.0 ppm. The upstream dissolved oxygen level was low (1.87 ppm) with a saturation of 23%, which is unsatisfactory for aquatic life. In addition, the mean dissolved oxygen level at the midstream site was only 0.98 ppm with a saturation of 12%, while downstream had the lowest dissolved oxygen level with 0.49 ppm and saturation of only 6%. In terms of electrical conductivity, the downstream site had the highest conductivity of 738.80 mS/cm, followed by the midstream site (642.33 mS/cm) and the upstream site (391.59 mS/cm). The recommended conductivity level for aquatic organisms is only 0.15–0.50 mS/cm.^12^ Total dissolved solids (TDS) are directly related with conductivity. The present study showed that TDS increased from upstream towards downstream. The oxidation reduction potential (ORP) was positive, but relatively low upstream (30 mV) and midstream (15 mV) and negative downstream (−190.75 mV). The recommended ORP limit for aquatic life is 300–500 mV.^13^,^14^ The biochemical oxygen demand for both upstream (13.22 ppm) and downstream (12.02 ppm) exceeded the recommended limit of 7.00 ppm by the DENR.^10^ The chemical oxygen demand level was higher at the downstream site (84 ppm) than the upstream site (20 ppm). Downstream exceeded the recommended chemical oxygen demand limit of 20 ppm.^15^ The nitrate level was low downstream (0.15 ppm) compared to upstream (0.56 ppm). For the phosphates, downstream (1.04 ppm) was high compared to upstream (0.42 ppm). The phosphates at the downstream site exceeded the recommended limit of 0.5 ppm.

**Table 2:** Mean Physico-Chemical Water Quality Variables of the Study Site and the Recommended Standards for Each Variable

**Variables**	**Upstream (Viente Reales)**	**Midstream (Cain gin bridge)**	**Downstream (McArthur bridge)**	**Recommended standard**
Visual color of water	Gray	Gray	Black	
pH	7.49 ± 0.05	7.28 ± 0.03	7.35 ± 0.01	6.5–9.0^10^
Temperature (°C)	28.03 ± 0.22	28.92 ± 0.53	30.75 ± 0.91	25–31 °C^10^
Dissolved oxygen (ppm)	1.87 ± 0.13	0.98 ± 0.15	0.49 ± 0.18	5.0 ppm^10^
Electrical conductivity (mS/cm)	391.58 ± 7.91	642.23 ± 4.62	738.80 ± 2.79	0.15–0.50 mS/cm^12^
Total dissolved solids (ppm)	249.55 ± 0	325 ± 2.15	481.40 ± 2.35	-
Oxidation reduction potential (mV)	30 ± 9.19	15 ± 4.56	−190.75 ± 4.99	300–500 mV^13^,^14^
Biochemical oxygen demand (ppm)	13.22 ± 1.95		12.02 ± 1.13	7 ppm^10^
Chemical oxygen demand (ppm)	20 ± 1.00	-	84 ± 3.00	20 ppm^15^
Nitrates (ppm)	0.56 ± 0.02	-	0.15 ± 0.02	7 ppm^10^
Phosphates (ppm)	0.42 ± 0.01	-	1.04 ± 0.01	0.5 ppm^10^

Aside from physico-chemical variables, the quality of the water samples was also determined through microbial indicators. The most common group of bacteria used for monitoring and assessing water quality are coliform bacteria. The coliform counts obtained at the upstream and downstream sites of the river were 3.5 × 10^4^ cfu/ml and 2.5 × 10^4^ cfu/ml, respectively. Based on the standards set by the United States Environmental Protection Agency (USEPA) for freshwater bodies of water that are used for recreational activities such as bathing, which is a criterion of Class C water, the water samples from the downstream and upstream sites exceeded the allowed limit of 126 cfu/ml.^16^ A bacterial count for water samples from the same sources was also obtained and the results showed that the bacterial counts obtained at the upstream and downstream locations of the river were 1.4 × 10^6^ cfu/ml and 7.5 × 105 cfu/ml, respectively.

### Heavy metal content of sediment

The results of the sediment quality analysis of heavy metals using X-ray fluorescence spectrometry and their assessment with different sediment quality guidelines are shown in Table 4. Lead had a value of 947 ppm at the upstream location and 783 ppm at the downstream site. Lead failed to satisfy the standards set by NOAA in which the severe effect level is 250.0 ppm.^11^ The midstream site had a Pb level of 391 ppm, which was relatively low compared to upstream and downstream. The Pb levels of the Meycauayan River were above the severe effect level of 250 ppm. A very high zinc (Zn) level was observed at the upstream (2157 ppm), midstream (2256 ppm) and downstream (741 ppm) locations. Both upstream and midstream exceeded the severe effect level of 820 ppm for Zn. The copper (Cu) levels were very high at the upstream location (877 ppm), followed by midstream (340 ppm) and lowest at the downstream site (134 ppm). However, Cu levels still exceeded the severe effect level of 110 ppm. Nickel (Ni) levels in the sediments were very high at the upstream (301 ppm) and midstream (178 ppm) locations and below the limit of detection at the downstream site. They also exceeded the severe effect level of 75 ppm. The manganese (Mn) level of the midstream and downstream sites was high and exceeded the severe effect level of 1100 ppm. The Cr level increased from upstream to downstream. It also exceeded the severe effect level of 110 ppm. The other heavy metals such as arsenic (As), mercury (Hg) and Cd were below the limit of detection.

**Table 3:** Microbiological Variables of the Upstream and Downstream Locations

**Parameter**	**Upstream (Viente Reales)**	**Downstream (McArthur bridge)**	**Recommended safe limits**
Total bacterial count (cfu/ml)	1.4 × 10^6^	7.5 × 10^5^	-
Total coliform count (cfu/ml)	3.5 × 10^4^	2.5 × 10^4^	126 cfu/100 ml^16^

**Table 4:** Heavy Metal Analysis of the Upstream, Midstream and Downstream Sites of the Meycauayan River

**Heavy metal**	**Upstream (Viente Reales)**	**Midstream (Caingin bridge)**	**Downstream (McArthur bridge)**	**NOAA (ppm)**^11^					

		*in mg/kg (ppm)*		TEC	TEL	LEL	PEC	PEL	SEL
Pb	947	391	783	35.8	35.0	31.0	128.0	91.3	250.0
As	< LOD	< LOD	< LOD	9.79	5.90	6.00	33.00	17.00	33.00
Hg	< LOD	< LOD	< LOD	0.18	0.17	0.20	1.06	0.48	2.00
Zn	2157	2256	741	121.0	123.0	120.0	459.0	315.0	820.0
Cu	877	340	134	31.6	35.7	16.0	149.0	197.0	110.0
Ni	301	178	< LOD	22.7	18.0	16.0	48.6	36.0	75.0
Mn	1047	1385	1695	-	-	460.0	-	-	1100.0
Cr	263	363	375	43.4	37.3	26.0	111.0	90.0	110.0
Cd	< LOD	< LOD	< LOD	0.99	0.59	0.60	4.98	3.53	10.0

Abbreviations: NOAA, National Oceanographic and Atmospheric Administration; TEC, threshold effect concentration; TEL, threshold effect level; LEL, lowest effect level; PEC, probable effect concentration; PEL, probable effect level; SEL, severe effect level.

## Discussion

The river water was gray at the upstream location and almost black downstream. This might be due to the color of sediments, which were gray to black in color, and the presence of pollutants. Temperature is an important indicator because it could have an effect on other physical phenomena, such as the rate of biochemical and chemical changes, reduction in solubility of gases and amplification of taste and odor of water.^17^ Temperature affects organisms present in the water since every living organism has its own tolerance range. It can affect other water quality variables such as dissolved oxygen, pH, ORP and conductivity. The pH level of the sites was slightly alkaline, which is desirable for aquatic organisms. Aquatic organisms function efficiently at a pH range of 6.5–9.0.

Dissolved oxygen is regarded as one of the most important indicators of water quality. It is essential for the survival and physiological function of fish and other aquatic organisms. A number of factors that could affect dissolved oxygen concentrations include water movement, presence of photosynthetic organisms (algae and aquatic plants) and bacteria, temperature and pollution. One cause of decreased dissolved oxygen concentration is pollution, due to effluents or runoff water with constituents that have high oxygen demand for decomposition.^18^ Based on the obtained results, the dissolved oxygen level did not meet the standard of the DENR, which is 5.0 ppm. The dissolved oxygen level was very low and unsatisfactory for aquatic life. It indicates that there is very low abundance of photosynthetic organisms in the river system due to pollution. In addition, the water on the upstream side was stagnant, which resulted in a decreased dissolved oxygen level. In terms of temperature level, dissolved oxygen is inversely related, and the solubility of gases decreases as temperature increases.^19^ The results agreed that a higher temperature site demonstrated relatively low dissolved oxygen levels. A decrease in dissolved oxygen level might also be due to the consumption of bacteria during decomposition. The Meycauayan River is classified as hypoxic, with a dissolved oxygen level < 2.0 ppm. During the assessment of the National Water Quality Status Report, the annual average dissolved oxygen in the Meycauayan River was 1.5 ppm (2003) and 1.2 ppm (2005).^18^ There were no changes in the mean dissolved oxygen levels in the river since 2003. In the latest National Water Quality Status Report (2006–2013), the Meycauayan River was regarded as “poor” water quality in terms of dissolved oxygen. Similarly, a very low dissolved oxygen level was observed in the Pasig River, in urban Manila. According to a study by Gorme *et al*., dissolved oxygen levels of the Pasig River were below the limit set by the DENR of 5.0 ppm from 1990 to 2009. Low dissolved oxygen levels were the result of the high discharge of domestic and industrial waste in the river system, similar to the Meycauayan River.^20^

Electrical conductivity levels are an indicator of the amount of salts and carbonates in water. Electrical conductivity is affected by the presence of inorganic dissolved solids such as chlorides, nitrate, sulfate, phosphate, sodium, magnesium, calcium, iron, and aluminum ions. This water quality parameter is an indicator that discharge, or other sources of pollution, have entered a water body.^21^ In the present study, electrical conductivity level increased from upstream to downstream. This could indicate that the downstream site is more polluted, as pollutants from the upstream site moved downstream and were accumulated. Freshwater streams require a conductivity level between 0.15 mS/cm to 0.50 mS/cm to support diverse aquatic life. Conductivity outside this range could indicate that the water is not suitable for certain species of fish or macroinvertebrates. Levels in industrial waters can range as high as 10 000 μS/cm.^12^ The conductivity level was alarmingly high and not suitable for aquatic life.

Another important water variable related to conductivity is TDS. Total dissolved solids are generally used as an aggregate indicator of the presence of chemical contaminants. Sources of TDS include agricultural runoff, leaching of soil contamination and point source water pollution from industrial or domestic waste.^18^ The presence of dissolved solids in water can affect the water balance of a cell. A low level of solids could cause swelling up of the cell as water enters it. On the other hand, the cell will shrink if the concentration is higher in the river because water inside the cell will move out. A high TDS level can affect an organism's ability to maintain proper cell density, making it difficult to keep its position in the water column. It might float up or sink down to a depth to which it is not adapted and might not survive.^22^ Sources of dissolved solids might come from different industrial activities and surface runoff along the river. There is no existing recommended level of TDS for rivers.

The ORP is a measurement that indicates the degree to which a substance is capable of oxidizing or reducing another substance. A positive reading indicates that water contains oxidizing agents, while a negative reading indicates that water contains reducing agents. In relation to dissolved oxygen, a high ORP level means that oxygen is abundant for the bacteria. The bacteria can decompose dead tissues and contaminants more efficiently. However, in the present study, the ORP values were low upstream and midstream and a negative ORP was observed downstream. In healthy waters, ORP values must be within 300 to 500 mV for aquatic life.^13^,^14^

Biochemical oxygen demand determines the amount of oxygen required for the decomposition of organic matter from a pollution source. The demand for oxygen does not occur directly where the effluent or runoff water is discharged, but is manifested somewhere downstream where decomposition finally occurs. Thus, a higher O value indicates more pollution.^18^ The results showed that river water exceeded the recommended limit of 7 ppm. Biochemical oxygen demand has an inverse relationship with dissolved oxygen. The higher the biochemical oxygen demand, the more rapidly oxygen is depleted in water. Hence, less oxygen is available to aquatic organisms. Different sources of biochemical oxygen demand in the river system might come from effluents from industries, wastewater treatment plants, domestic waste and water runoff. The DENR-Environment Management Bureau reported that the annual biochemical oxygen demand level of the Meycauayan River in 2003 was 38.2 ppm and increased in 2005 to 119.8 ppm.^18^ Based on the latest National Water Quality Status Report, the biochemical oxygen demand of the Meycauayan River is still regarded as “poor” by the DENR-Environment Management Bureau.^2^ The Pasig River was also severely polluted with a biochemical oxygen demand level exceeding the limit of 7.0 ppm from 1990 to 2009. The sources of biochemical oxygen demand in the Pasig River were domestic, industrial and solid wastes.^20^

Chemical oxygen demand is a measure of the capacity of water to consume oxygen during the decomposition of organic matter and oxidation of inorganic chemicals such as ammonia and nitrite. Chemical oxygen demand is the total measurement of all chemicals (organic and inorganics) in the water. Chemical oxygen demand values are always greater than the biochemical oxygen demand measurements. The recommended limit for chemical oxygen demand is 20 ppm.^15^ Chemical oxygen demand has an impact on dissolved oxygen as high levels decrease the amount of oxygen available for aquatic organisms. A hypoxic environment can reduce cell functioning, disrupt balance and could result in the death of aquatic organisms.

Nitrates and phosphates in excess amounts can cause significant water quality problems. This can accelerate eutrophication, causing a dramatic increase in aquatic plant growth and changes in the type of plants and animals living in the river. This, in turn, affects dissolved oxygen, temperature, and other water quality indicators. Excess nitrates can cause hypoxia or low levels of dissolved oxygen and can become toxic to aquatic organisms at higher concentrations (10 mg/l).^23^ The results of the present study indicate that nitrate levels did not exceed the limit of 7 ppm. Phosphate levels were high downstream. Phosphates are usually present in low concentrations in the environment. An increase in phosphate levels is due to natural and human sources. These include soil and rock leaching, wastewater treatment plants, runoff from fertilized lawns and cropland, failing septic tanks, runoff from animal manure, drained wetlands, and household waste.^24^

Aside from the physico-chemical variables, water sample quality was also determined through microbial indicators. The most common group of bacteria used in monitoring and assessing water quality are coliform bacteria. Coliforms are non-spore-forming, rod-shaped bacteria which are commonly found in the environment and in the feces of warm-blooded organisms. Due to the characteristics of coliforms, they are used in detecting human and animal fecal contamination in bodies of water. The World Health Organization uses coliforms as a microbiological parameter for assessing water quality due to high occurrence of the bacteria in the feces of humans and warm-blooded animals, high counts in wastewater and polluted waters and absence from pure water and other environments which do not have any contact or intervention with humans and other animals.^25^ Coliform count is one of the most important water quality indicators related to human health.

### Heavy metal content of sediments

Heavy metal contamination enters through different pathways either from point sources such as discharge of industrial wastes or non-point sources from runoff of landfills.^26^ These metals are high priority pollutants because of their relatively high toxicity, persistence and ability to bioaccumulate in aquatic biota.^27^ Heavy metals trapped in aquatic environments tend to accumulate in sediments which act as sinks and sources of contaminants in water. Heavy metals have a severely damaging effect on the ecological balance of the recipient environment and tend to bioaccumulate in the food chain and ultimately reach human consumers.^28^ There are no sediment standards available for the Philippines, however there is an available standard developed by NOAA, the Screening Quick Reference Table for Inorganics in Sediment.^11^ This guide categorizes heavy metal levels as on the threshold, probable or severe effect level. The data of heavy metals in sediments indicate that the Meycauayan River is severely polluted with Pb, Zn, Cu, Mn and Cr. This might be due to the industries located along the river. Informal industries such as Pb-acid battery recycling could be contributing to the high Pb levels in river sediments. One possible source of high Cr might be leather tanning industries in Meycauayan.

Existing studies have monitored heavy metals in water, but not in sediments in the Philippines. It is essential that sediment quality be considered to determine overall environmental health because of the importance of benthic organisms. A study was conducted to determine the heavy metal concentration of sediments from Manila Bay and its inflow rivers. The Meycauayan River discharges into Manila Bay. The study showed that Pb, Cd, Zn and Cu were elevated in riverine sediments.^29^ Few studies have addressed sediment heavy metal pollution in river quality assessments in the Philippines.

### Statistical analysis

The results of one-way analysis of variance (ANOVA) for water quality shows significant differences in variables such as pH, temperature, dissolved oxygen, conductivity, total dissolved solids, chemical oxygen demand, nitrates and phosphates. The post-hoc Tukey test showed significant differences for these variables across the three study sites. In the analysis of sediment quality, one-way ANOVA and post-hoc Tukey test showed significant differences in sediment for Pb, Zn, Cu, Mn and Cr across the three study sites.

## Conclusions

The results of the present study indicate the presence of severe heavy metal pollution in sediments. Lead, Zn, Cu, Mn and Cr exceeded the severe effect level by NOAA for the three stations. One-way ANOVA showed significant differences (p < 0.001) in sediment heavy metal concentrations across the three sites. The deterioration of the Meycauayan River has been a result of rapid industrialization, urbanization and population growth.

The present study provides preliminary information on the severity of pollution in the Meycauayan River. Hence, a further, more comprehensive study of the Meycauayan River is recommended to determine its environmental health status. A water quality index assessment would help to determine which water quality variable should be prioritized. Further studies should include all of the abiotic and biotic elements of the river system. In addition, sediment quality index assessment must be included and highlighted because of the importance of sediment to the health of surrounding water bodies. This will provide a better vision of the current status of the Meycauayan River. The present study could be used as a basis for the development of strategies for reviving, rehabilitating and protecting the Meycauayan River.
